# In this month’s Bulletin

**DOI:** 10.2471/BLT.18.000618

**Published:** 2018-06-01

Nabil Karah et al. (370) call for better provision of dialysis services to Syrian refugees in Lebanon. Maggie Montgomery et al. (371) point out that cholera prevention requires adequate water and sanitation in all settings.

Tatum Anderson (374–375) reports on the need for a global response to an number of increased yellow fever outbreaks. Dixon Chibanda explains to Fiona Fleck (376–377) how he developed the Friendship Bench approach to mental health care in Zimbabwe. 

## Burkina Faso

### Detecting tuberculosis contacts

Giorgia Sulis et al. (386–392) implement prevention for exposed children.

## Malawi

### Preventing a water-borne disease

Maurice M’bangombe et al. (428–435) examine the use of oral cholera vaccine.

## Uganda

### Trauma prevention and research

Abdulgafoor M Bachani et al. (423–427) present the results of a postgraduate training course.

## Global

### Monographs for essential medicines 

Lukas Roth et al. (378–385) identify gaps in public pharmaceutical standards.

### Making decisions around universal health coverage

Mark G Shrime et al. (393–401) present a data envelopment analysis adapted to the health system. 

### How much Zika virus?

Michelle M Haby et al. (402–413) review evidence for prevalence estimates of asymptomatic infection.

### Another angle on the sustainable development goals

Valerie A Luyckx et al. (414–422) show how all goals can be connected to kidney disease prevention and treatment. 

### Consigning mercury to the past

Julian Fisher et al. (436–438) outline steps towards the phase down of dental amalgam.

### Beyond national borders

Bettina Borisch et al. (439–440) provide an update on the Global Charter for the Public’s Health.

